# Applying gene flow science to environmental policy needs: a boundary work perspective

**DOI:** 10.1111/eva.12393

**Published:** 2016-07-08

**Authors:** Caroline E. Ridley, Laurie C. Alexander

**Affiliations:** ^1^ National Center for Environmental AssessmentU.S. Environmental Protection Agency, Office of Research and DevelopmentWashingtonDCUSA

**Keywords:** boundary work, environmental policy, gene flow, genetically engineered crops, watershed connectivity

## Abstract

One application of gene flow science is the policy arena. In this article, we describe two examples in which the topic of gene flow has entered into the U.S. national environmental policymaking process: regulation of genetically engineered crops and clarification of the jurisdictional scope of the Clean Water Act. We summarize both current scientific understanding and the legal context within which gene flow science has relevance. We also discuss the process by which scientific knowledge has been synthesized and communicated to decision‐makers in these two contexts utilizing the concept of ‘boundary work’. Boundary organizations, the work they engage in to bridge the worlds of science, policy, and practice, and the boundary objects they produce to translate scientific knowledge existed in both examples. However, the specific activities and attributes of the objects produced varied based on the needs of the decision‐makers. We close with suggestions for how scientists can contribute to or engage in boundary work with policymakers.

## Introduction

Many in the scientific community are interested in producing work that successfully informs decision‐making, from traditional applied research for site‐specific management decisions to broader synthetic work for addressing larger scale environmental issues. The study of gene flow and its role in evolutionary and ecological processes has contributed to important national and regional policies in the United States. Below, we briefly recount some of the major advances in gene flow science while highlighting policies that the science has informed. Next, we introduce the processes by which scientific information becomes or does not become a part of environmental management or policy decisions, which are worthy of study themselves and can take a variety of forms. We chose the concept of ‘boundary work’ to capture the ways that scientists and policymakers can work across disciplinary boundaries to solve social–environmental problems. Then, we bring together gene flow science, its relevance to particular (sometimes intricate) policies, and the activities and products of boundary work in two in‐depth examples. We close with suggestions for how scientists can contribute to or engage in boundary work with policymakers.

### Role of gene flow in evolutionary and ecological processes

Gene flow is the collective term for mechanisms resulting in gene movement between populations of the same or different species or subspecies, and it is the evolutionary force that genetically coheres populations (Slatkin 1985). Conversely, lack of gene flow attributable to spatial, temporal, or behavioral isolation allows for genetic divergence between populations over time, via local adaptation or genetic drift. For example, restricted gene flow between populations of Pacific salmon (*Oncorhynchus* spp.) that differ in habitat requirements and spawning timing and/or location has resulted in divergence (e.g., Tallman 1994; Wood and Foote 1996; Wenburg et al. 1998; Hendry and Day 2005). Understanding and documenting this divergence has been key to the protection of ‘evolutionarily significant units’ (Moritz 1994; Pennock and Dimmick 1997; Waples 1998a) of Pacific salmon under the U.S. Endangered Species Act, the criteria for which are (i) reproductive isolation and (ii) contribution to the evolutionary legacy of the species (Waples 1991).

Isolation and small population size can combine to decrease genetic diversity through drift, leading to inbreeding effects such as those that have been observed in populations of some threatened and endangered species (Ellstrand and Elam 1993; Keller and Waller 2002). Gene flow to genetically augment or ‘rescue’ these populations can contribute to recovery in fitness and population size (Vila et al. 2003; Hogg et al. 2006; Pimm et al. 2006). The combined demographic and genetic effects of migration and gene flow can ensure the existence of populations that would otherwise fail to be self‐sustaining (Lenormand 2002; Cosentino et al. 2012; Mushet et al. 2013). Evidence of gene flow in metapopulations fragmented by loss of habitat patches (e.g., conversion of wetlands to agricultural land uses) has provided insights into species’ shifts to the use of complementary habitats, such as ditches (Favre‐Bac et al. 2016).

The interaction of gene flow with adaptive evolution is complex. On the one hand, gene flow of sufficient magnitude relative to the strength of selection can limit the evolution of adaption (Lenormand 2002; Garant et al. 2007; but see also Waples 1998b). This principle has been key in developing the refuge strategy to swamp the evolution of resistance to *B.t*. toxins used for the control of agricultural pests, with some success and some failure (Gould 1998; Tabashnik et al. 2005, 2008; Gassmann et al. 2011; Farkas 2015). On the other hand, gene flow can provide the genetic variation upon which selection can act. Creation or maintenance of corridors promoting gene flow and strategies for the intentional translocation of preadapted individuals (assisted gene flow) have received renewed attention as tactics for conserving species under climate change; the hope is that gene flow will result in the spread of adapted genotypes and/or the maintenance of genetic diversity, which will enable populations to adapt to future climate conditions (Lankau et al. 2011; Aitken and Whitlock 2013; Christie and Knowles 2015; Nicotra et al. 2015).

Increased availability of genetic markers and improved statistical approaches for their analysis have led to a much better understanding of the roles of historical and ongoing migration and gene flow in the structuring of populations within species (Manel et al. 2005; Ellstrand 2014). Landscape genetics is another recent advance to integrate genetic patterns, evolutionary and ecological processes, and spatial determinants of organismal movement (Manel et al. 2003; Manel and Holderegger 2013). Landscape genetics of species in stream networks and its utility in explaining the distribution and persistence of species diversity has had recent application to the protection of water resources under the U.S. Clean Water Act (see our second example below).

Gene flow between populations of different species or subspecies, also termed hybridization, has both evolutionary and ecological consequences. Issues that can arise in hybrids (e.g., reduced fitness, sterility) can have consequences for the persistence of species or subspecies, especially when one taxon is relatively rare (Ellstrand and Elam 1993; Rhymer and Simberloff 1996). Early research championed interspecific gene flow as a creative force for adaptation. For example, Anderson (1949), Stebbins (1959), and Lewontin and Birch (1966) believed that interspecific hybridization provided essential variation on which selection could act. A more contemporary view confirms the role that hybridization can play in adaptive introgression, but also acknowledges the development of stable hybrid zones, homoploid and allopolyploid hybrid speciation, colonization of novel ecological niches, and evolution of invasiveness as outcomes of interspecific gene flow (Schwenk et al. 2008; Schierenbeck and Ellstrand 2009; Soltis et al. 2014; Yakimowski and Rieseberg 2014). Understanding the rates and outcomes of hybridization has been important for the regulation of genetically engineered crops (see our first example below).

### Boundary work and development of influential scientific information

The goal of boundary work is to facilitate effective communication across organizational and disciplinary boundaries, such as those that exist between science and policymaking (Guston 2001). Boundary work formalizes procedures for interactions between scientists and nonscientist decision‐makers (Gieryn 1983). Organizations dedicated to boundary work at the interface of science, policy, and practice include agricultural cooperative extension services in the United States (Brugger and Crimmins 2015) the United Nations Environment Programme DTU (Technical University of Denmark) Partnership, formerly the UNEP Risø Center (Lee et al. 2014), and the Ecosystem‐Based Management Tools Network (www.ebmtools.org). Boundary organizations can also be virtual organizations comprised of members from participating groups (Crosby et al. 2009).

To facilitate information transfer, successful boundary work involves creation and use of ‘boundary objects’. Examples of boundary objects include conceptual models, maps, reports, or contractual agreements. A risk assessment is one specific kind of boundary object that is developed within the well‐known and accepted paradigm of risk‐based decision‐making (National Research Council 1983, 2009); risk assessments use scientific information to characterize the nature and magnitude of health or ecological risks and ultimately inform decisions by risk managers.

An important part of constructing and managing interfaces between communities of practice is clear identification of what kind or quality of information each community considers *useful* (Clark et al. 2002, 2011; Cash et al. 2003). However, the potential for scientific information to influence policy also depends on the user's perception that the information and the process that produced it are valid and trustworthy. Clark et al. (2002) proposed the term *influential information* and identified three attributes that make information influential, rather than just potentially useful. Here, we apply the attributes specifically to boundary objects:

1. Saliency: Do decision‐makers perceive the boundary objects as relevant to their policy needs, questions, or choices? Are they timely?
2. Credibility: Do decision‐makers perceive that the evidence and arguments in the boundary objects meet standards of scientific plausibility and adequacy? Are the boundary objects of sufficient quality and quantity?
3. Legitimacy: Do decision‐makers perceive the process that produced the boundary objects as unbiased, respectful of differing value systems, and fair in the treatment of opposing views?

Standards for evaluating these three attributes can be context‐dependent and subjective. This can be frustrating for scientists, who value objectivity. In addition, these attributes are interrelated, so attempts to strengthen one attribute may result in unwanted trade‐offs among the others (Clark et al. 2002). For example, to avoid perception of bias in a risk assessment, the assessment could be conducted by scientists having no direct communication with policymakers. This arrangement may increase the assessment's perceived legitimacy, but may decrease its salience (e.g., if the scientists framing the assessment misinterpret policy needs or fail to recognize policy constraints on its scope). In contrast, a risk assessment conducted entirely within a policymaking organization would benefit from a better understanding of policy needs (increased salience) but could be subject to real or perceived bias toward a particular policy outcome (decreased legitimacy). Thus, an important function of boundary work is to develop strategies for balancing trade‐offs among these attributes (Clark et al. 2002).

Further introduction to boundary work can be found in the study by Mollinga (2010), who provides a framework for using boundary work to deal with the complexities inherent in natural resources management research.

## Boundary work and gene flow science: Examples of knowledge and process

In this section, we introduce two examples of how gene flow science has influenced and could continue to influence decision‐making under existing U.S. federal laws. We provide brief background on the scientific issues and policy context, followed by descriptions of the boundary work processes and approaches used at the science–policy interface. Throughout, we apply the concept of boundary work as a way of understanding and interpreting the various activities and products (i.e., the boundary objects) generated, highlighting where processes have the potential to impart key attributes (*saliency, credibility, legitimacy*) to decision‐relevant scientific information. These examples do not represent the full range of possible applications of gene flow science to policy development under these or other laws or the full range of processes by which scientists and decision‐makers interact; they also are not meant to provide ‘good’ or ‘bad’ examples of how these processes work. They do illustrate real‐world boundary work at the interface of science and policy and shed light on the complexities inherent to that process.

### Example 1: gene flow and genetically engineered crops

#### Science background

Gene flow between crops, their progenitors, and wild or weedy relatives is a well‐documented phenomenon. Genetic and/or morphological evidence indicates that 22 of the world's 25 most important crops exchange genes with free‐living relatives (Ellstrand 2003). Often, this evidence comes from places where the crop and its relatives are both native, but there are examples of gene exchange in locations where both the crop and a compatible relative are introduced (e.g., radish/wild radish [*Raphanus sativus* L.*/R. raphanistrum* L.] and sorghum/johnsongrass [*Sorghum bicolor/S. halepense* L.] in the United States (Morrell et al. 2005; Hegde et al. 2006)). Occasionally, an introduced crop has the capacity to exchange genes with a native relative, as in the case of upland cotton (*Gossypium hirsutum* L.) and the native Hawaiian cotton (*G. tomentosum* Nutt. ex Seem.) (Ellstrand 2003; Pleasants and Wendel 2010).

The consequences of gene exchange with crops, in terms of the genetic composition, phenotypic characteristics, and demography of populations of introgressed wild relatives or hybrid populations, are variable and can be difficult to predict. They depend on many factors, including gene flow dynamics (e.g., frequency, duration), characteristics of crop and wild populations (e.g., size, diversity), crop–wild hybrid traits, and the environment (Ellstrand et al. 2013; Mercer et al. 2014; Hooftman et al. 2015). In general, consequences of highest concern occur when populations of wild relatives are genetically or demographically pushed to extinction (Ellstrand and Elam 1993; Lu 2013) or when hybridized populations become weedy or invasive (Ellstrand et al. 2010; Roso et al. 2010). Although these two dramatic outcomes are rarely observed, the persistence of crop genes in populations of wild relatives has been documented in over a dozen systems (Ellstrand et al. 2013), with emerging evidence for introgression in additional systems (Dangl et al. 2015). Beyond population‐level effects, the impacts of gene exchange on community‐level interactions (e.g., trophic effects, predator–prey dynamics) are difficult to generalize and will depend on many system‐ and site‐specific variables.

Based on empirical evidence from nontransgenic crop‐to‐wild relative gene flow, there is clearly the potential that a transgene could move into wild populations, establish, and spread over generations. Theory and modeling confirm this (Hooftman et al. 2007; Ellstrand et al. 2013; Garnier et al. 2014). In one study that modeled consistent, low‐level gene flow from a crop to a wild relative, even crop alleles that were disfavored had the potential to reach fixation (Haygood et al. 2003). Studies have uncovered presence of transgenes in wild or weedy plant populations (Watrud et al. 2004; Reichman et al. 2006; Schafer et al. 2011; Wegier et al. 2011; Greene et al. 2015). In at least one case, it appears that a cultivated transgenic plant (creeping bentgrass) exchanged genes with individuals outside of cultivation (Reichman et al. 2006). Many open questions remain, such as whether transgenes in wild populations will persist and whether (and in what situations) transgenes established in populations of wild and weedy relatives will affect population‐ and community‐level dynamics.

#### Policy background

The movement of transgenes into wild or weedy populations is of interest in the policy arena, because genetically engineered crops and their potential environmental and economic effects are examined by the U.S. government under existing law and regulations. Under the Coordinated Framework for Regulation of Biotechnology (U.S. OSTP 1986, 1992), products of genetic engineering are regulated by three U.S. government agencies: the Food and Drug Administration, the Department of Agriculture, and the Environmental Protection Agency (EPA). The Federal Insecticide, Fungicide, and Rodenticide Act (FIFRA) is the current law under which the EPA evaluates genetically engineered crops that express pesticidal substances (referred to as plant‐incorporated protectants); we do not consider other laws administered by other federal agencies here.

Under FIFRA, the EPA is directed to examine all adverse environmental effects of registering a pesticide for use in the United States. If data show that such effects are not caused by a pesticide, the EPA registers it for sale, distribution, and use. Cultivation of crops that have been genetically engineered to produce a pesticidal substance to protect them from insects (e.g., Cry proteins from *Bacillus thuringiensis*) essentially introduces a pesticide into the environment. Novel exposures of the pesticidal substance outside the cultivated environment could arise if there is gene flow of plant‐incorporated protectants to wild or feral populations of sexually compatible plants. In principle, the results of such exposures could range from negative effects on nontarget, susceptible insects, to altered population dynamics of sexually compatible plants, to no discernable environmental effects. Therefore, when a crop genetically engineered with a plant‐incorporated protectant is being examined by the EPA for potential adverse effects under FIFRA, gene flow with wild relatives is one consideration (Wozniak and Martinez 2011).

#### Boundary work processes

Within the EPA's Office of Chemical Safety and Pollution Prevention, scientists in the Office of Pesticide Programs develop approaches for collecting and analyzing gene flow data as part of the risk assessment process for plant‐incorporated protectants. This includes engaging with risk managers to determine what data they require to make a decision about registration of a plant‐incorporated protectant for sale and use. The role of the risk manager is to address the policy question: Will the use of a pesticide have ‘unreasonable adverse’ effects? In contrast, the role of scientists and the scientific information they generate (as synthesized and communicated by the boundary organization) is to identify all possible effects, without regard to what might be an ‘unreasonable adverse’ effect in a policy context.

The work of the boundary organization (i.e., the EPA's Office of Pesticide Programs) begins with communication from potential applicants who would like to register a plant‐incorporated protectant or receive an experimental use permit (which makes possible smaller scale cultivation of crops genetically engineered with a plant‐incorporated protectant for research purposes). The boundary organization meets with the potential applicant to discuss the data needs of the risk manager to formulate a decision *(saliency)*. The assessment of potential environmental risk associated with gene flow of a plant‐incorporated protectant is requested on a case‐by‐case basis. Environmental risk includes both exposure and hazard, defined as the probability of gene flow and the consequences of gene flow, respectively. After this meeting, the applicant summarizes its understanding of the data that they must supply, and the boundary organization either agrees or suggests changes based on the needs of the decision‐maker.

The boundary organization works with independent scientists to ensure that their approaches are sound and that information it communicates to decision‐makers is complete and accurate *(credibility)*. Written into FIFRA is a mechanism to provide EPA with independent scientific advice on health and environmental safety issues related to pesticides: the FIFRA Scientific Advisory Panel (U.S. EPA 2014). It is a standing panel of seven members that are nominated by the National Institutes of Health and the National Science Foundation and serve 4‐ to 6‐year terms. Panel members’ expertise is augmented by the Food Quality Protection Act Science Review Board, which consists of scientists that can serve on an *ad hoc* basis to help the Scientific Advisory Panel perform its duties. Nominations for the Science Review Board come from many sources, including the public *(legitimacy)* (U.S. EPA 2004). Scientists chosen to serve as *ad hoc* panel members are selected for specific expertise on issues being considered by the panel, helping to ensure the credibility of the panel and the reports it produces.

When a decision‐relevant scientific issue arises, the boundary organization (i.e., Office of Pesticide Programs) crafts a series of charge questions to be answered by the Scientific Advisory Panel. These questions are distributed to the panel and published in the Federal Register. The questions are discussed by the panel members in a public meeting (at which comments by the public are also accepted), and a report responding to these questions is published on the EPA website some months afterward. Scientific Advisory Panel reports can be considered boundary objects, as they consist of information that is designed to be salient to a decision but do not represent decision objectives themselves.

Since 2000, the EPA has twice requested scientific reviews or information about gene flow from the Scientific Advisory Panel (Table 1). In 2000, the panel reviewed a draft reassessment of the environmental effects of *B.t*. crops registered at the time. This boundary object (called a Biopesticide Registration Action Document) included an extensive review of available data about gene flow in corn, potato, and cotton, including data supplied by registrants, publicly available literature, and results of workshops and seminars *(credibility)*. The panel also responded to questions (Table 1) about the adequacy of proposed approaches to quantifying environmental risks of crossing between a plant‐incorporated protectant‐containing crop and wild or feral relatives (FIFRA Scientific Advisory Panel 2001), particularly in terms of the possibility and probability of gene flow. The boundary organization revised its document in response to the Scientific Advisory Panel suggestions and to public comments, and a final version was released (U.S. EPA 2001). One of the major conclusions from the document was that ‘there is no significant risk’ of gene flow from *B.t*. corn, potato, or cotton to wild or weedy relatives in the United States or its possessions or territories, with the exception of cotton in Hawaii, Florida, and the Caribbean. The conclusion was based on evidence that wild or weedy relatives are either absent from areas of cultivation or reproductively isolated from *B.t*. crops via phenology or chromosomal incompatibilities. Thus, risk managers no longer request data on gene flow for these crops in the ‘no significant risk’ regions. EPA has prohibited sale or distribution of *B.t*. cotton seed in locations in Puerto Rico, Hawaii, the U.S. Virgin Islands, and Florida where wild relatives exist because of the potential for gene flow and a lack of information about its consequences.

**Table 1:** Gene flow‐related charge questions that were posed to the Federal Insecticide, Fungicide, and Rodenticide (FIFRA) Scientific Advisory Panel in 2000 and 2009

Scientific Advisory Panel 2000	Scientific Advisory Panel 2009
Does quantifying risk (e.g., hybridization rates, gene introgression) provide adequate means to assess potential environmental impact and determine approval of a plant pesticide which has wild or feral relatives in the United States? If yes, what further risk assessment is warranted to evaluate the risk of outcrossing?	The EPA asks the Panel to discuss whether it is possible to evaluate, in part, impacts of a gene flow event by gathering data on target (pest) species which are associated with the wild species (transgene recipient).
Are isolation distances as proposed for certified or registered seed considered sufficient to mitigate gene flow between *B.t*. crops and wild or feral populations of sexually compatible species? If not, what distances or measures should be imposed to mitigate outcrossing?	The EPA asks the Panel to discuss whether the gathered data will allow estimating the degree to which resistance to these target species may influence the population dynamics or invasiveness of the wild relative.
Does the panel agree that the gene flow and outcrossing assessment contained in the background documents are adequate for the currently registered *B.t*. crops? If not, what additional data or issues should be considered to assess gene flow and outcrossing risks from *B.t*.‐expressing plant products?	The EPA asks the Panel to discuss whether empirical data regarding the target species (e.g., fungi, insects) and nontarget species (e.g., pollinators, detritivores) associated with the sexually compatible wild relative have the potential to inform about risks to the [sexually compatible wild relative] population and the associated community.
	The EPA asks the Panel to discuss whether an understanding of the potential effect(s) of introgressed transgenes on basic plant habit, phenology and physiology provide a basis for assessing potential impacts following a gene flow event.

In 2009, another Scientific Advisory Panel convened, and the questions focused on potential *outcomes* of gene flow and understanding what information would be necessary to understand environmental impacts, assuming that gene flow was known to occur (FIFRA Scientific Advisory Panel 2009) (Table 1). Such input should be valuable if and when risk managers are asked to consider the registration or experimental use of plant‐incorporated protectants in which the crop species has already been shown to exchange genes with wild relatives (e.g., canola and sunflower); besides cotton, such a request has not been made to date.

### Example 2: gene flow and watershed connectivity

#### Science background

Connectivity is a foundational concept in the science and management of aquatic ecosystems. A recent EPA assessment reviewed the literature on the hydrologic, biogeochemical, and biological connectivity of small or temporary streams, nontidal wetlands, and other ‘upstream’ source waters to ‘downstream’ rivers, lakes, estuaries, and coastal seas (U.S. EPA 2015). The goal of this assessment was to synthesize evidence from multiple fields of aquatic connectivity research, ranging from geomorphology and hydrology to landscape ecology and evolution, to inform rulemaking on the scope of federal protections for surface waters under the U.S. Clean Water Act (see policy background below; Alexander 2015). Here we focus on a few illustrative examples of connections formed by dispersal and gene flow between populations inhabiting both ‘upstream’ (e.g., nontidal stream and inland wetland) and ‘downstream’ (e.g., river, lake, and marine) environments.

Dispersal is defined as the movement of individuals or propagules with potential for gene flow (Ronce 2007). Dispersal strategies reflect species’ responses to past and present selective pressures, and can depend on environmental conditions and the phenotype of individuals (Kisdi et al. 2012; Starrfelt and Kokko 2012). For example, active annual upstream migrations of anadromous salmon that connect marine and freshwater habitats along the entire length and breadth of a river network are timed to coincide with favorable conditions for spawning, which vary by population (e.g., Hodgson and Quinn 2002). Despite capability for such long‐distance movement, natal imprinting and local adaptation have produced genetically and ecologically differentiated groups within and among species of Pacific salmon (*Oncorhynchus* spp.) that are connected by limited dispersal of ‘straying’ adults (Waples 1991; Tallman 1994; Wood and Foote 1996; Wenburg et al. 1998; Waples et al. 2004; Hendry and Day 2005). More widespread dispersal does occur, for example, in juvenile Pacific salmon taking advantage of rearing habitats that were reconnected by dam removal (Anderson et al. 2013). Passive dispersal of aquatic organisms can also be spatially extensive, with local and regional factors such as episodic or seasonal variations in streamflow or prevailing winds being important factors controlling direction, distance, timing, and rate (Gornall et al. 1998; Figuerola et al. 2005; Nilsson et al. 2010). Animal vectors, including migratory water fowl, have been shown to transport large numbers of wetland plants, macroinvertebrates, and zooplankton (or their propagules) up to 1400 km (Mueller and van der Valk 2002). Even when relatively rare, such long‐distance dispersal events can have important effects on metapopulation survival and genetic diversity (Bohrer et al. 2005).

Aquatic macroinvertebrates and fish have been studied and modeled to characterize rates and geographic patterns of gene flow and the consequences for population‐level genetic diversity, persistence, and adaptation (Bohonak and Jenkins 2003; Waples et al. 2004; Gustafson et al. 2007; Chaput‐Bardy et al. 2009). For example, Whiteley et al. (2010) found that barriers to gene flow between upstream and downstream populations of coastal cutthroat trout (*Oncorhynchus clarkii clarkii*) promoted genetic divergence within streams and loss of genetic diversity in small, upstream populations. Additionally, asymmetric gene flow was observed in some streams, with greater upstream‐to‐downstream dispersal of individuals (Whiteley et al. 2010). Asymmetric gene flow can increase overall genetic diversity in low‐dispersing species by infrequent transfer of rare alleles from locally adapted populations (Chaput‐Bardy et al. 2009). Dispersal and gene flow also have contemporary and future repercussions for community integrity and resilience—that is, the community's ability to absorb stresses, retain function, and adapt to new conditions (Sgro et al. 2011). Corridors that facilitate dispersal and gene flow across fragmented landscapes can counteract the effects of loss of genetic diversity via drift (e.g., Christie and Knowles 2015). Maintaining the adaptive capacity of keystone species through conservation of genetic diversity could be especially important for resilience of entire communities (Nicotra et al. 2015).

Advances have been made toward predictive modeling of the distribution of genetic variation between populations by accounting for the spatial arrangement of suitable habitat (Fagan 2002; Hughes et al. 2009; Morrissey and de Kerckhove 2009; Paz‐Vinas et al. 2015). For instance, in dendritic stream networks, the distributions of genetic and species diversity (and, thus, future evolutionary trajectories and community composition, respectively) have been linked to spatially influenced, historical processes of migration and gene flow which tend to be highly constrained by physical stream structure (Hughes et al. 2009; Finn and Poff 2011; Finn et al. 2011; Paz‐Vinas et al. 2015). With climate change and increasing pressure from habitat loss and fragmentation, information about gene flow will play an increasingly important role in the ability of resource managers to develop practices that enhance the capacity of individuals, populations, species, and communities to cope with their new environments (Crook et al. 2015).

#### Policy background

The goal of the 1972 U.S. Clean Water Act is to ‘restore and maintain the chemical, physical, and biological integrity of the Nation's waters’. For its first three decades, the Clean Water Act protected almost all surface waters, including small or temporary streams, rivers, all types of wetlands, lakes, reservoirs, and coastal seas. Throughout its history, numerous legal challenges to this law have sought to limit federal regulation of surface waters through a narrow interpretation of its scope (Adler 2015). Legal debate came to a head in 2006 when the U.S. Supreme Court issued a split (4‐1‐4) opinion, with no clear majority, in response to litigation challenging the jurisdiction of the Clean Water Act over wetlands and streams having only seasonal or ephemeral connections to ‘navigable’ waters. A key outcome of this court case was the judicial opinion that federal protections for streams and wetlands depend on the existence of a ‘significant nexus’ with ‘navigable waters in the traditional sense’ (*Rapanos v. United States*). Thus, despite the ambiguous court ruling, the central role of connectivity emerged as a *policy*‐relevant consideration for evaluating federal stream and wetland protection in the United States.

In 2015, the administering agencies (EPA and the U.S. Army Corp of Engineers) published new regulations known as the Clean Water Rule that clarified the scope of the Clean Water Act in light of the Court's rulings. The Clean Water Rule establishes categories of waters that meet legal requirements for jurisdiction, and a definition of ‘significant nexus’ based on scientific understanding of the connectivity of water bodies, that is, the functions and pathways by which streams and wetlands affect the chemical, physical, or biological integrity of downgradient waters (Alexander 2015).

#### Boundary work processes

Boundary work on this effort began with a request to the EPA's Office of Research and Development (the agency's science office) from the EPA's Office of Water (the agency's water policy office) to summarize and synthesize available scientific evidence on the functional relationships between different types of water bodies. Here, the boundary work is best characterized as iterative dialogues between the scientists conducting the assessment and the decision‐makers developing the policy. The functions of a boundary organization were performed by designated individuals within the science and policy offices who identified and documented the roles, needs, and capabilities of both organizations. For the scientists, this step was critical for understanding the types and quality of scientific evidence required by the decision‐makers (*saliency and credibility*), and for obtaining policy input on the purpose and scope of the assessment (*legitimacy*). For the decision‐makers, this step was needed to understand what kinds of questions were answerable with scientific evidence (*saliency*); the strengths and limits of scientific knowledge (*credibility*); and the information quality standards for the assessment (*legitimacy*). Over a series of meetings, the two parties established the information needs (the policy questions), the scope of the scientific assessment (the science questions; U.S. EPA 2015), the timeline for delivery of draft and final reports (boundary objects), and the processes for peer review and public input, as required by EPA policy (U.S. EPA STPC 2015). This series of intensive dialogues, which took place over a period of three months, exemplifies one of the most important functions of boundary work. Because decision‐makers and scientists had different lexicons and realms of expertise, a translation process was needed to develop a clear understanding of the policy needs, the role of science, and the assessment goals. This process is akin to the problem formulation step of risk assessment (National Research Council 2009). The documented goals of the scientific assessment to address decision‐maker needs were as follows:

1. Provide a conceptual framework for understanding watershed connectivity from a systems perspective;
2. Synthesize evidence of pathways and functions by which streams and wetlands might affect chemical, physical, and biological integrity of downgradient rivers, lakes, and coastal waters;
3. Identify climate and landscape factors that influence connectivity;
4. Inform the identification of categories of waters, based on strength and effects of connectivity; and
5. Apply the resulting framework and evidence to case studies of different water body types.

Given the broad, national scope of the policy decision, the legitimacy component of boundary work was particularly important in this example. To ensure legitimacy, three separate peer reviews of the scientific assessment (boundary object) were conducted during its development: (i) reviews of early chapter drafts by experts who were selected for their knowledge of specific topics or ecosystem types; (ii) a review of the entire draft assessment by a multidisciplinary panel of 11 experts, organized and managed by an independent contractor; and (iii) a review of the revised assessment by the EPA's Science Advisory Board, established in 1978 as directed by the U.S. Congress to provide scientific advice to the EPA Administrator. While different in both structure and function, the FIFRA Scientific Advisory Panel (in Example 1) and the EPA Science Advisory Board both provide a public process for objective review (*legitimacy*) of ‘the quality and relevance of the scientific and technical information being used by the EPA or proposed as the basis for Agency regulations’ (*credibility* and *saliency*; http://yosemite.epa.gov/sab/sabpeople.nsf/WebCommittees/BOARD).

To improve transparency and public input, nominations for Science Advisory Board panelists are solicited from the public *(legitimacy)*. Nominees are evaluated on relevant expertise and lack of perceived or actual conflicts of interest *(credibility)*. The Science Advisory Board held five public meetings over a 10‐month period and released four drafts before publishing their final report of consensus comments (another boundary object). The EPA assessment was revised in response to comments from the panel and the public. The completed scientific assessment incorporated more than 1200 sources of peer‐reviewed scientific information (U.S. EPA 2015). In a separate meeting, the chartered Science Advisory Board considered the adequacy of the scientific information provided by the EPA report and peer‐review process as a scientific basis for the proposed regulation (U.S. EPA SAB 2014b).

The Science Advisory Board did not specifically comment on the role of gene flow science in establishing or understanding biological connectivity among water bodies. However, their consensus was that biological connectivity is essential to aquatic ecosystem integrity, and the panel did encourage the EPA to more strongly emphasize biological connectivity in the conceptual framework and review of evidence. Their report included an appendix of suggested literature to strengthen the assessment of dispersal, recruitment, and ecological integration of aquatic habitats (U.S. EPA SAB 2014a).

## Discussion

In this article, we show that gene flow science is relevant to several areas of policy development at the EPA. There are similarities and differences between the two examples in terms of the type of scientific information applicable to decision‐making, the nature of the decision itself, and the attributes of the boundary organizations and boundary objects involved (Table 2).

**Table 2:** Summary of the policy and science contexts for the genetically engineered crops and watershed connectivity examples presented in this study, as well as their boundary organization and boundary object attributes

	Genetically engineered crops	Watershed connectivity
Relevance of gene flow science	Gene flow between crops engineered with a plant‐incorporated protectant and wild or weedy relatives is possible and could result in environmental effects.	Connectivity in aquatic and semiaquatic species connects populations and could affect population‐ and community‐level attributes, including composition and resiliency.
Policy context	EPA examines potential environmental effects of pesticides, including plant‐incorporated protectants, under the Federal Insecticide, Fungicide, and Rodenticide Act (FIFRA).	Clean Water Act has been interpreted by the courts to consider evidence of ‘significant nexus’ with navigable waters.
Boundary organization	EPA Office of Chemical Safety and Pollution Prevention and Office of Pesticide Programs	Members of EPA Office of Research and Development and Office of Water
Boundary organization attributes		
Mediated boundary	Engaged across the boundary between crop gene flow scientists and decision‐makers within EPA who determine data requirements for plant‐incorporated protectant registrants.	Engaged across the boundary between research scientists (hydrologists, ecologists, etc.) and decision‐makers within the regulatory agencies who interpret the legislative and judicial direction on Clean Water Act jurisdiction.
Boundary objects created	Synthesis of scientific understanding of gene flow between corn, potato, cotton, and their respective wild relatives (U.S. EPA 2001), and two reports by the Scientific Advisory Panel (FIFRA Scientific Advisory Panel 2001, 2009) requested by the boundary organization.	Assessment of scientific understanding of connectivity of water bodies written by the boundary organization (U.S. EPA 2015), and two peer‐review reports of that assessment requested by the boundary organization, one by an independent peer‐review panel and one by the Science Advisory Board (U.S. EPA SAB 2014aa).
Boundary object attributes		
Saliency	Synthesis—Interactions between boundary organization and decision‐makers.	Assessment—Approach determined through iterative interactions between boundary organization and decision‐makers.
	Scientific Advisory Panel reports—Include answers to a set of charge questions, posed by the boundary organization, that are presumably relevant to decision‐maker needs.	Peer‐review reports—Comprised of a set of charge questions posed by the boundary organization.
Credibility	Synthesis—Authored by scientists, synthesis incorporated sources of data ranging from data submitted by plant‐incorporated protectant registrants to publicly available literature and was reviewed by the Scientific Advisory Panel.	Assessment—Authors had applicable scientific training, the assessment incorporated over 1200 published scientific articles and was reviewed by multiple groups of independent scientific experts.
	Scientific Advisory Panel reports—Authors are required to have subject matter expertise and must disclose financial interests and other potential conflicts of interest.	Peer‐review reports—Authors are required to have subject matter expertise and must lack real or perceived conflict of interest.
Legitimacy	Synthesis—Incorporated comments submitted by the public.	Assessment—Released for public comment when reviewed by the Science Advisory Board. When final document was released, it was accompanied by a ‘response to comments’ document, clarifying how panel and public comments were incorporated.
	Scientific Advisory Panel reports—Panel members may be nominated by the public. Members of the public are allowed to address the panel during public meetings.	Peer‐review reports—Science Advisory Board panel members are nominated by the public. All panel deliberations are open to the public, and members of the public are invited to provide verbal comments to the panel during meetings.

The issues related to gene flow and regulation of plant‐incorporated protectants outlined in our first example are very specific in nature, because gene flow is directly related to data necessary to assess potential environmental effects of plant‐incorporated protectants and statutory responsibilities under FIFRA. The issue of environmental effects of plant‐incorporated protectants is quite broad and not limited to potential effects from gene flow (U.S. EPA 2001), yet gene flow remains of specific interest, especially in cases where a crop genetically engineered with a plant‐incorporated protectant will be grown in proximity to wild or weedy relatives. This contrasts with the very broad issue of the connectivity of aquatic systems that was needed to support clarifying the jurisdictional scope of the Clean Water Act. Gene flow, although not of specific interest to decision‐makers, was one of many kinds of evidence contributing to an overall understanding of system connectivity. Gene flow provides important evidence of how aquatic systems are bound together by biological connections that can affect the biological integrity of surface waters at multiple spatial and temporal scales.

The process by which boundary organizations operated at the interface between decision‐makers and independent scientific advisors showed similarities. In both examples, it was easy to identify each of the interacting groups (boundary organization versus decision‐maker versus scientific advisors). The existence of distinct groups is important for differentiating roles and maintaining the integrity of processes on either side of the science–policy boundary. The identifiable groups engaged in boundary work in both cases actively dialoged with decision‐makers in an iterative way, consulted with independent scientific experts, and created boundary objects.

The number of scientists engaged in boundary work and the number of scientists engaged in the panels of independent scientific experts were considerable in both examples, although especially so for the connectivity case. They worked together to achieve saliency, credibility, and legitimacy of the scientific information provided to decision‐makers. However, boundary organizations and independent scientific experts served very different roles in this process. As reviewers cannot participate in developing the boundary object and authors cannot participate in reviewer deliberations about it, their communications are defined by the need for independence on both sides (boundary integrity) as well as for clear translation of needs (boundary integration). Independent experts themselves created what could be considered boundary objects in the form of reports that summarized answers to sets of charge questions posed by the boundary organizations.

Occasionally, boundary organizations must balance all or some of the three attributes (saliency, legitimacy, credibility) to utilize input from independent experts in the synthesis of information used for decision‐making. In 2009, gene flow experts on the FIFRA Scientific Advisory Panel took the initiative to answer questions *not* directly posed to them in an effort to provide information that they believed was salient (FIFRA Scientific Advisory Panel 2009). This may be warranted, especially if there are recent, significant scientific advances of which the boundary organization or decision‐makers are not aware. Another possibility is that independent experts may misunderstand some aspect of the decision‐making process (e.g., limits of legal authorities, timeline constraints on a decision). There is also a chance that decision‐makers will perceive an agenda or bias that ultimately affects the legitimacy of the panel if they answer questions not posed to them. Boundary work in the future related to this issue will have to balance saliency and legitimacy to incorporate expert feedback and provide scientific information to decision‐makers. In the connectivity example, the Science Advisory Board panel favored more strongly emphasizing the role of biological connectivity via migration of waterfowl, which may not be as salient as other kinds of evidence, due to a U.S. Supreme Court ruling that rejected use by migratory waterfowl as the sole basis for Clean Water Act regulation of wetlands. The boundary organization was thus faced with balancing saliency and credibility when revising their assessment in response to the Science Advisory Board review.

There were also differences in boundary work processes and the boundary objects between our examples. Both examples explicitly included iterative communication, but it differed in duration. Communication with decision‐makers extended over a period of nearly five years in the watershed connectivity example, compared with two years in the example of genetically engineered crops. There was a very high requirement set by the decision‐makers for saliency, credibility, and legitimacy of the information contained in the connectivity assessment, resulting in lengthy processes of scientific synthesis, public outreach, economic analysis, and policy development. The Biopesticide Registration Action Document was also a boundary object with considerable length and scope, produced using approaches to ensure its saliency, credibility, and legitimacy, but the needs of the decision‐makers may have been on a narrower timeline than in the connectivity example. This demonstrates that the processes of boundary work and attributes of boundary objects must meet the needs and expectations of the parties involved. No one process is likely to be most effective or necessarily more transparent (Cook et al. 2013).

For scientists that are interested in contributing to boundary work at the science–policy interface, we have several recommendations, which vary in their time investment and their proximity to the science–policy interface. First, boundary work often necessitates summarizing and synthesizing large bodies of existing scientific research. Because of this, a high‐quality, published literature review that has already compiled and interpreted the body of work on a subject is valuable. Although a single review is unlikely to encompass everything needed for a specific policy application, it does leapfrog the process forward. What constitutes a ‘high‐quality’ review in this context? Reviews published in well‐respected journals are traditionally considered high‐quality; transparency in the methodology (e.g., search strategies, criteria for inclusion or exclusion of studies) is also becoming increasingly important. Systematic reviews are common in the medical literature and are just catching on more broadly in other scientific disciplines as a way to ensure objectivity and credibility. Reviews that are conducted with a clear and documented methodology are most likely to be useful in boundary work.

Second, scientists can join government institutions that do boundary work. Opportunities exist to be engaged directly in boundary work and to produce policy‐relevant science as employees, fellows, advisors, or collaborators at all levels of government. It is also our experience that scientists involved in boundary work rely on networks of collaborators, as well as the scientific literature, to bring the appropriate expertise to bear in policy‐relevant topics. For academic researchers who are interested in boundary work, maintaining professional relationships with colleagues *already* engaged in boundary work is an easy way to become indirectly connected to the science–policy interface.

Third, scientists with demonstrated policy‐relevant expertise can choose to serve on one of the many scientific advice panels to the federal government such at the FIFRA Scientific Advisory Panel or EPA's Science Advisory Board (e.g., Blockstein 2002). There are many opportunities to self‐nominate to these independent, expert panels and the time committed is usually finite.

Finally, many communities of practice now exist that consider boundary work one of their primary missions. The National Socio‐Environmental Synthesis Center (www.sesync.org), the National Center for Ecological Analysis and Synthesis (www.nceas.ucsb.edu), and the Powell Center for Analysis and Synthesis (powellcenter.usgs.gov) each provide the opportunities to scientists to collaborate in transdisciplinary ways and to synthesize science for use by decision‐makers.
